# NLRC5/CITA expression correlates with efficient response to checkpoint blockade immunotherapy

**DOI:** 10.1038/s41598-021-82729-9

**Published:** 2021-02-05

**Authors:** Sayuri Yoshihama, Steven X. Cho, Jason Yeung, Xuedong Pan, Gregory Lizee, Kranti Konganti, Valen E. Johnson, Koichi S. Kobayashi

**Affiliations:** 1 Department of Microbial Pathogenesis and Immunology, Texas A&M Health Science Center, 415A Reynolds Medical Building, College Station, TX 77843 USA; 2 Department of Gastroenterology and Nephrology, Graduate School of Medicine, Chiba University, Chiba, 260-8670 Japan; 3 Department of Immunology, Graduate School of Medicine, Hokkaido University, Kita 15, Nishi 7, Kita-ku, Sapporo, 060-8638 Japan; 4 Department of Statistics, Texas A&M University, College Station, TX 77843 USA; 5 Departments of Melanoma Medical Oncology and Immunology, The University of Texas MD Anderson Cancer Center, Houston, TX 77054 USA; 6 Texas A&M Institute for Genome Science and Society, Texas A&M University, College Station, TX 77843 USA

**Keywords:** Immunosurveillance, Tumour biomarkers

## Abstract

Checkpoint blockade-mediated immunotherapy is emerging as an effective treatment modality for multiple cancer types. However, cancer cells frequently evade the immune system, compromising the effectiveness of immunotherapy. It is crucial to develop screening methods to identify the patients who would most benefit from these therapies because of the risk of the side effects and the high cost of treatment. Here we show that expression of the MHC class I transactivator (*CITA*), *NLRC5*, is important for efficient responses to anti-CTLA-4 and anti-PD1 checkpoint blockade therapies. Melanoma tumors derived from patients responding to immunotherapy exhibited significantly higher expression of *NLRC5* and MHC class I-related genes compared to non-responding patients. In addition, multivariate analysis that included the number of tumor-associated non-synonymous mutations, predicted neo-antigen load and *PD-L2* expression was capable of further stratifying responders and non-responders to anti-CTLA4 therapy. Moreover, expression or methylation of *NLRC5* together with total somatic mutation number were significantly correlated with increased patient survival. These results suggest that *NLRC5* tumor expression, alone or together with tumor mutation load constitutes a valuable predictive biomarker for both prognosis and response to anti-CTLA-4 and potentially anti-PD1 blockade immunotherapy in melanoma patients.

## Introduction

Checkpoint blockade immunotherapy has emerged as one of the most promising strategies to treat patients with various cancers^1,2^. Although many successes in single or combinatory use of anti-CTLA-4, PD-1 or PD-L1/2 antibodies have been documented in a variety of malignancies, responses are only typically observed in a minority of patients for any given regimen^3,4^. Considering the substantial risk of autoimmune side effects and the high cost of the treatment^5–7^, it is critical to develop screening methods to identify the subsets of patients who would most benefit from these therapeutics. While immunohistochemistry for PD-1/PD-L1 has been approved by the FDA, neo-antigen load, copy number alterations, TCR sequencing, multi-parameter flow cytometry, SERPINB mutations or nCounter gene expression profiling have been assessed for predicting responses to checkpoint blockade therapies, their predictive power and usefulness as potential biomarkers are limited^8–11^.

Successful cancer growth and progression relies on the combination of both suppression and evasion of the host immune system^12^. One of the major mechanisms of immune suppression in the tumor microenvironment is through impaired effector T cell function and can occur through multiple mechanisms such as increased expression of inhibitory immune checkpoint molecules, immunosuppressive enzymes and recruitment of immunosuppressive immune cells^13^. Similarly, immune evasion from CD8+ T cells through defects in the MHC class I-mediated antigen presentation pathway is a common occurrence^14–16^, with MHC class I loss or reduction reported in lung cancer (93%), prostate cancer (91%), cervical cancer (90%), pancreas cancer (86%), breast cancer (84%), colorectal cancer (78%), among others^14,17–21^. Impaired MHC class I may manifest through many mechanisms such as loss of heterozygosity, gene mutations, epigenetic suppression or most importantly, downregulation at the transcriptional level^14^. Indeed it has recently been shown that the MHC class I transactivator (CITA), *NLRC5*^22,23^ is a major suppression target to facilitate immune evasion in multiple cancers^24^. CITA/NLRC5 is an IFN-γ-inducible nuclear protein^25–27^ and transcriptionally regulates MHC class I gene activation via a CITA enhanceosome complex that specifically associates with MHC class I gene promoters^26,28,29^. As a master transcriptional activator of the MHC class I antigen presentation pathway, CITA/NLRC5-dependent MHC class I genes include HLA-A,-B,-C,-E,-F as well as the immunoproteasome component *LMP2* (*PSMB9*), peptide transporter *TAP1* and β2-microglobulin (*B2M*)^23,26,29,30^. The functional consequences of *NLRC5* deficiency in vivo constitutes failure of effective immune responses, accompanied with increased susceptibility to pathogen infection due to reduced constitutive and inducible expression of MHC class I gene expression and subsequently poor CD8+ T cell activation^31–35^. On the other hand, overexpression of NLRC5 in cancer models has demonstrated improved tumor immunogenicity^36^. In human cancers, genetic and epigenetic changes in *NLRC5* gene are associated with impaired expression of MHC class I and related genes and reduced activity of CD8+ cytotoxic T cells^24^. Strikingly, increased expression of *NLRC5* is highly associated with improved 5-year survival of patients with skin, rectal, bladder, uterine, cervical or head/neck cancers, thus showing *NLRC5* expression is potentially valuable as a prognostic marker^37^.

## Results

### The expression of NLRC5 and MHC class I associated genes are correlated with response to anti-CTLA-4 antibody therapy

**Figure 1:**
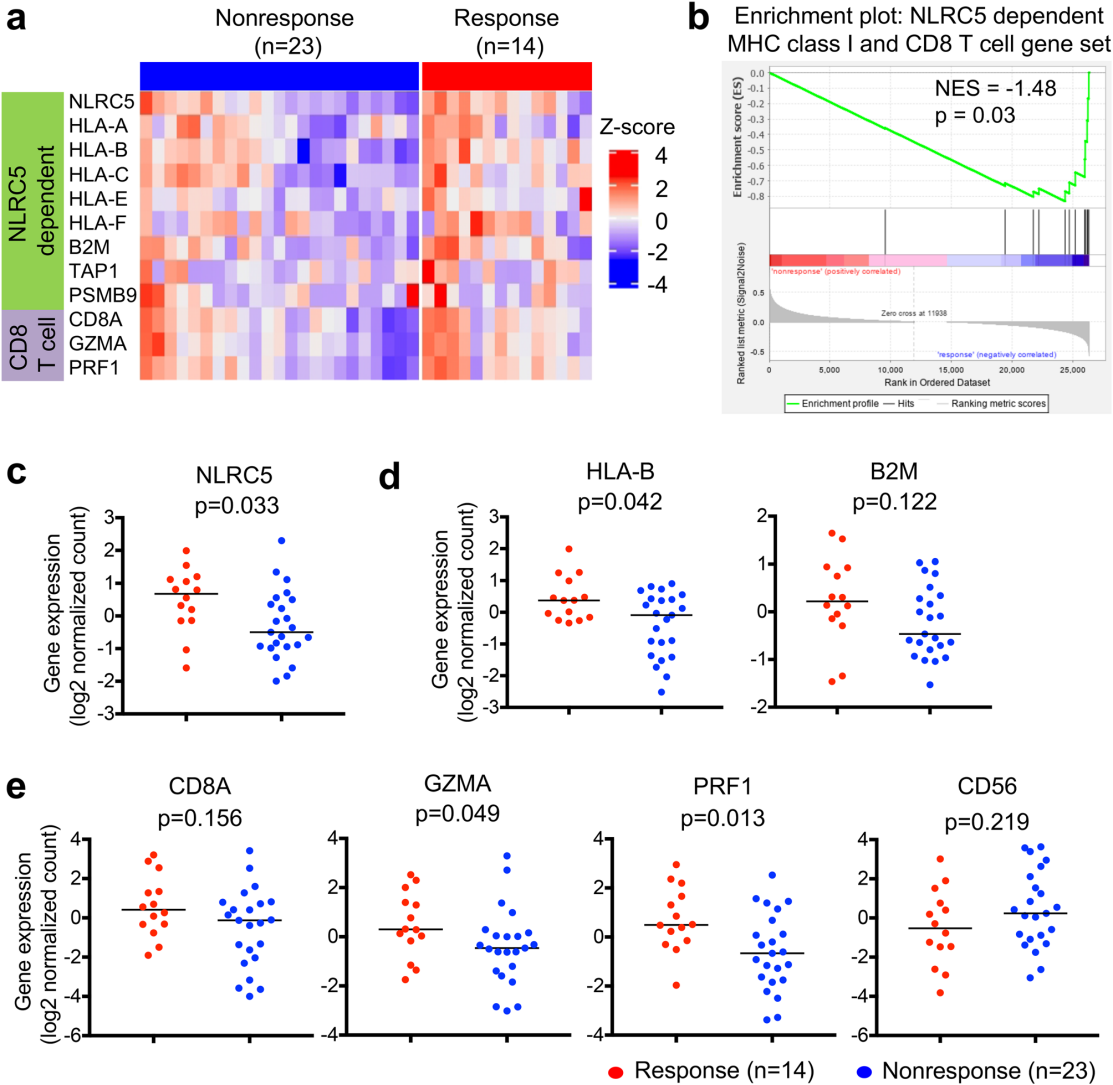
The expression of NLRC5-dependent MHC class I and CD8+ T cell genes are correlated with response to anti-CTLA-4 antibody therapy. Patients groups who benefitted from anti-CTLA4 antibody therapy (Response, n = 14) and who did not (Nonresponse, n = 23) were analyzed for differential gene set enrichment by (**a**) heatmap and (**b**) GSEA as well as individual gene expression levels of (**c**) *NLRC5*, (**d**) *HLA-B*, *B2M*, (**e**) *CD8A*, granzyme A (*GZMA*), perforin (*PRF1*) and *CD56*. Bar represents the median value. P-values calculated using Mann–Whitney U test. NES, normalized enrichment score.

Since *NLRC5* expression is required for efficient cytotoxic CD8+ T cell responses, we hypothesized that *NLRC5* may be important for mediating the clinical benefits of cancer patients treated with checkpoint inhibitors^24,37^. We analyzed and compared the gene expression level of *NLRC5* and its dependent genes in melanoma between the groups who benefitted from the treatment (responder) and who did not benefit (non-responder). Among the melanoma patient cohort who received anti-CTLA-4 checkpoint blockade therapy, we observed a reduction in the gene expression level of NLRC5-dependent MHC class I and CD8+ T cell genes in non-responders versus responders (Fig. 1a). Gene set enrichment analysis indicated that this NLRC5-dependent MHC class I and CD8+ T cell gene set was upregulated in responders (Fig. 1b). Among these, we found that *NLRC5* expression was significantly elevated in the group who benefitted from the anti-CTLA-4 therapy (Fig. 1c). Because of the role of NLRC5 as a major regulator of MHC class I and related genes, the expression of *NLRC5* is correlated with *HLA-B* and *B2M* in various cancers^24^ as well as in this melanoma patient cohort (Fig. S1). In addition to NLRC5, the responder group exhibited higher expression of *HLA-B* than the non-responder group, and *B2M* showed a similar trend although it was not statistically significant with this cohort size (Fig. 1d). NLRC5 is required for optimal recruitment and activation of CD8+ cytotoxic T cells in cancers^24,37^. As expected, the expression of *NLRC5* in various cancers^24^ and in this melanoma cohort was also correlated with the expression level of markers for CD8+ T cell activation, *CD8A* and granzyme A (*GZMA*)/perforin (*PRF1*), but not *CD56*, a marker for NK cells (Fig. S1). The responder group exhibited higher expression of *GZMA* and *PRF1* (Fig. 1e). Although *GZMA* and *PRF1* are expressed in both CD8+ T cells and NK cells, the high expression of *GZMA* and *PRF1* was likely due to activated CD8+ T cells rather than NK cells, since *CD56* expression in the responder group was not significantly different than that of the non-responder group (Fig. 1e). These data suggest that *NLRC5* and *NLRC5*-mediated MHC class I dependent CD8+ T cell activation is important for effective response to anti-CTLA-4 checkpoint blockade immunotherapy.

### *NLRC5* expression and load of mutation or neoantigen provide predictive information for the response to anti-CTLA-4 therapy

**Figure 2:**
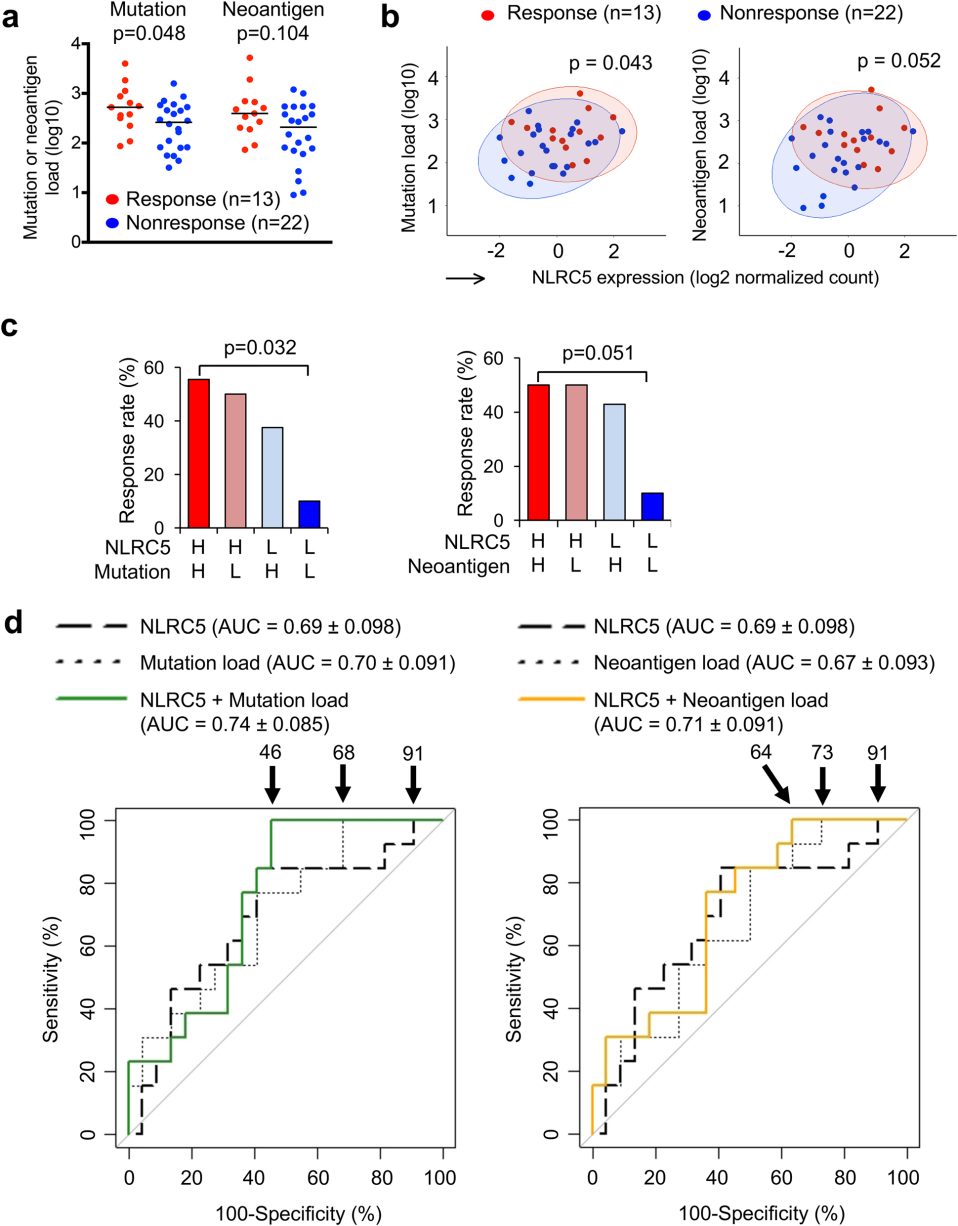
Multivariate analysis with *NLRC5* expression and load of mutation or neoantigen provide predictive information for the response to anti-CTLA-4 therapy. (**a**) Comparison of mutation and neoantigen load between response (n = 13) and non-response (n = 22) groups. P-values were calculated using Mann–Whitney U test. (**b**) Scatterplots for *NLRC5* expression and mutation or neoantigen load. 95% confidence ellipses about the centroids were drawn for both response (red circle) and non-response group (blue circle). P-values were calculated using Hotelling’s Test. (**c**) Response rate to anti-CTLA-4 therapy in the four groups stratified by *NLRC5* expression and mutation/neoantigen load. Cohort was divided into four groups based on the level of *NLRC5* expression and mutation or neoantigen load. The response rate (%) to the therapy among each group was calculated. Patients carrying higher value of the median are defined as high group (H), those carrying lower value of the median are defined as low group (L) in respective variables. Statistical significance between the groups of high *NLRC5* expression/high mutation or neoantigen load and low *NLRC5* expression/low mutation or neoantigen load were determined by the χ2 test. (**d**) ROC curves for logistic regression models using the respective combination of *NLRC5* expression, mutation load and neoantigen load. The numbers with arrow are showing false positive rate with 100% sensitivity. AUC (area under the curve) ± SE (standard error) is depicted.

It has been shown recently that neoantigen load is an important predictor of responses to anti-CTLA-4 therapy; high neoantigen loads in melanoma are correlated with favorable responses to anti-CTLA-4 therapy^38^. Similarly, the number of total mutations (mutation load), which has been demonstrated to be highly correlated with neoantigen load in various cancers as well as samples in this cohort (Fig. S2), is important in predicting response^38,39^. In order to test if the addition of mutation/neoantigen load to *NLRC5* expression would improve predictions, we performed multivariate analysis by logistic regression treating these variables as covariates. Consistent with a previous report^38^, responding patients in this study also showed higher neoantigen load and number of tumor-associated mutations (Fig. 2a). Scatter plots for *NLRC5* expression combined with neoantigen load or mutation number showed non-responder groups were clearly separated from responders (Fig. 2b). Patients were then stratified by *NLRC5* expression and neoantigen load or number of mutations, yielding four groups (high/high, high/low, low/high, and low/low). The response rate in the group with low *NLRC5* expression and low neoantigen load (or low mutation number) was significantly less than that of the group with high *NLRC5* expression and high neoantigen load (or high mutation number) (Fig. 2c). These results suggest that two variables, *NLRC5* expression and neoantigen load (or mutation number) may be useful to jointly identify non-responders. ROC analysis based on the prediction equation from logistic regression showed a substantial increase in the area under the curve (AUC) when mutation/neoantigen load was included as a predictor in the regression model (Fig. 2d). For the model that included *NLRC5* and mutation load, 100% sensitivity was obtained at a 46% false positive rate (Fig. 2d, left). Without mutation load, a false positive rate of 91% was required to achieve 100% sensitivity. Similarly, false positive rate with 100% sensitivity was improved to 64% when neoantigen load was included as a predictor (Fig. 2d, right). These data further indicate that analysis with two variables are useful to predict the patient population who will not respond to anti-CTLA-4 therapy.

### Combination of *PD-L2* expression with *NLRC5* expression and mutation or neoantigen load are sensitive predictors for responses to anti-CTLA-4 therapy

**Figure 3:**
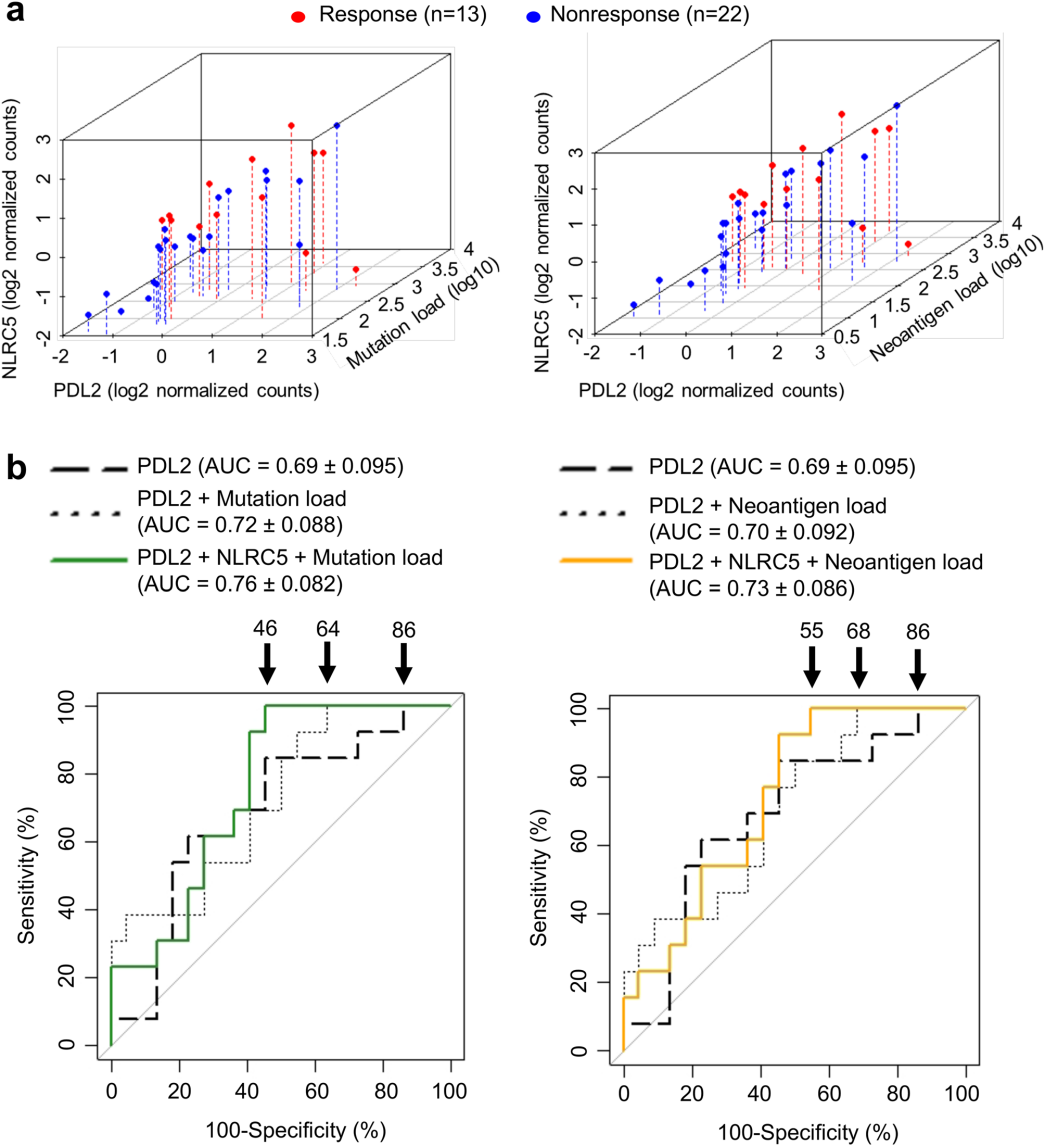
Combination of *PD-L2* expression with *NLRC5* expression and mutation or neoantigen load are sensitive predictors for responses to anti-CTLA-4 therapy. (**a**) Scatterplots for *NLRC5* and *PD-L2* expression with mutation load (left panel) or neoantigen load (right panel) for response (n = 13) and nonresponse (n = 22) groups. (**b**) ROC curves for logistic regression models using the respective combination of *PD-L2* expression, *NLRC5* expression, mutation load and neoantigen load. The numbers with arrow are showing false positive rate with 100% sensitivity. AUC (area under the curve) ± SE (standard error) is depicted.

Based on the superior performance of our ROC curve analyses using NLRC5 and mutation or neoantigen load (Fig. 2d), we sought to further improve prediction by adding more variables. We reasoned that *CTLA-4*, *PD-1*, *PD-L1* or *PD-L2* might be good candidates because their expression has been proposed to be correlated with responses to checkpoint blockade immunotherapies^38,40,41^; therefore, we first examined the correlation between *NLRC5* expression and the expression of these genes. The expression of *NLRC5* exhibited intermediate to high correlation with the expression of *CTLA-4* (Pearson’s correlation coefficient 0.70) and *PD-1* (0.83), while the correlation between *NLRC5* expression and expression of *PD-L1* (0.44) and *PD-L2* (0.54) was lower (Fig. S3), suggesting that *CTLA-4* and *PD-1* might not be good predictive variables to partner with *NLRC5*. ROC curve analyses revealed that the AUC was the highest when *NLRC5* expression, mutation load and *PD-L2* expression were included as covariates (Table S1). Scatter plots with *NLRC5* expression, *PD-L2* expression and mutation load/neoantigen load suggests that a part of the non-responder group did not overlap with the responder group (Fig. 3a, Supplementary video1-8). ROC curve analysis using these variables showed improvement of the false positive rate, which decreased from 86% using the single variable (*PD-L2* expression) to 46% or 55% using three variables (*PD-L2*, *NLRC5* expression and mutation load or neoantigen load, respectively) (Fig. 3b). This analysis suggests that the combination of three variables are also useful in identifying the patient population that does not respond to anti-CTLA-4 therapy.

### Combination of *NLRC5* expression and load of mutation or neoantigen provide prognostic information for the response to anti-CTLA-4 therapy

**Figure 4:**
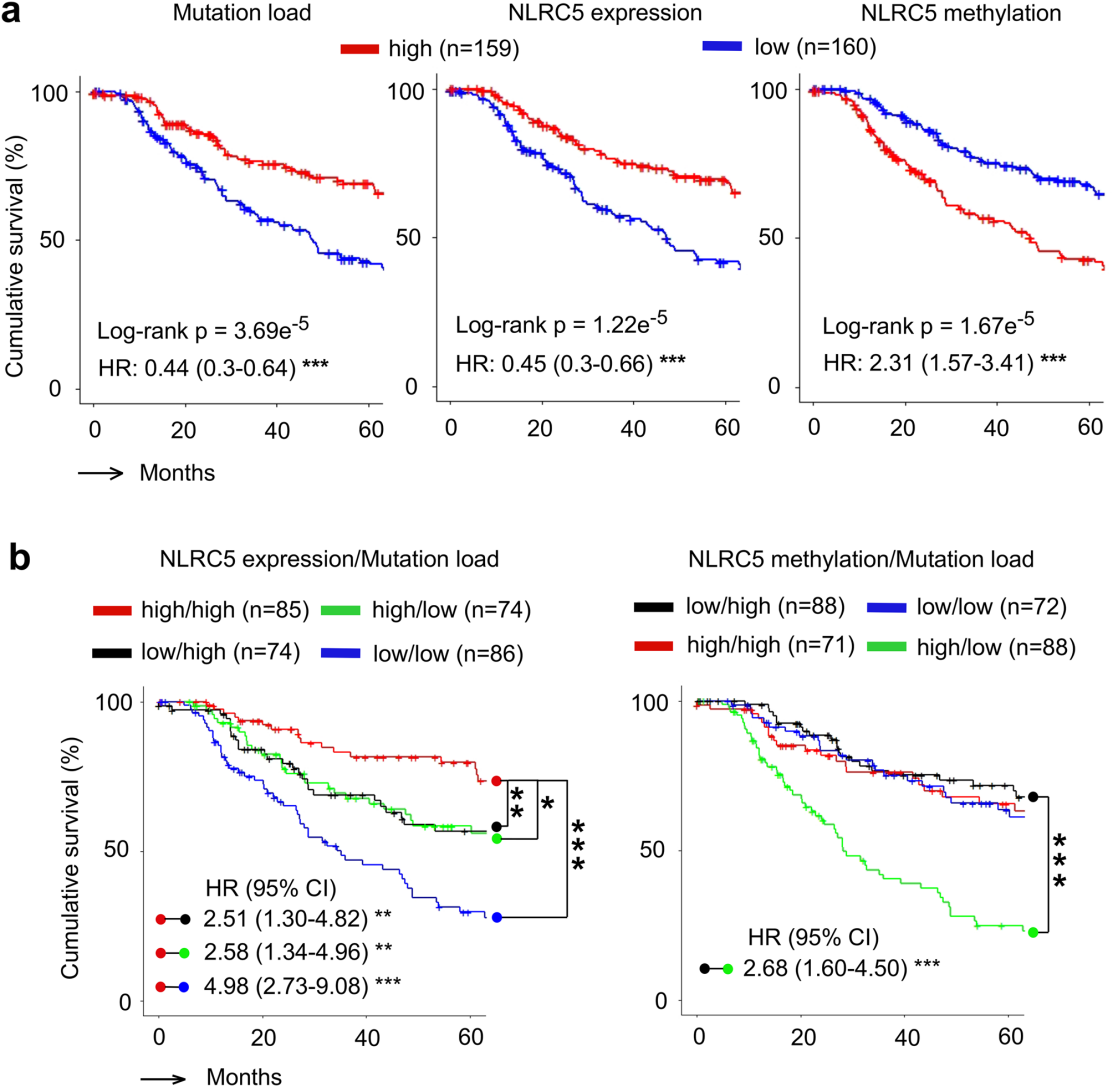
Combination of *NLRC5* expression and load of mutation or neoantigen provide prognostic information. (**a**) Kaplan–Meier estimates of five year overall survival of patients with high and low mutation load (Left), *NLRC5* gene expression (Middle), and *NLRC5* methylation (Right). Patients in the TCGA melanoma cohort were stratified by medians into high and low groups (n = 159 and n = 160). (**b**) Kaplan–Meier estimates of five year overall survival of patients with varying levels of two factors, *NLRC5* expression and mutation load (Left) and *NLRC5* methylation and mutation load (Right). Patients were stratified by two factors (*NLRC5* expression/*NLRC5* methylation and mutation load) in a similar fashion with (**a**), yielding four groups (high *NLRC5* expression/*NLRC5* methylation and high mutation load, likewise, high and low, low and high, low and low). Pairwise log-rank test was used to analyze the survival in indicated pairs. Hazard ratio (HR) and 95% confidence interval (CI) was determined by multivariate analysis using Cox regression model (see Methods). *p < 0.05; **p < 0.01; ***p < 0.001.

Previously it was shown that *NLRC5* expression is correlated with prognosis of patients with multiple cancer types^24^. The multivariable logistic regression including *NLRC5* expression together with mutation load or neoantigen load indicated that the analysis of two variables would be superior to predict responses to anti-CTLA-4 checkpoint blockade therapy (Fig. 2b–d). Since these variables are critical for immune surveillance against cancer, we hypothesized that an association would be observed with patient prognosis and overall survival. Using melanoma patient data from the TCGA database, we performed a survival curve analysis using Kaplan–Meier estimates for overall survival and a multivariate Cox proportional hazards model for hazard ratios. The cohort was divided into two groups based on values higher or lower than the median for mutation load, *NLRC5* expression and *NLRC5* promoter methylation. The high mutation patient group demonstrated better prognosis than the low mutation group (HR = 0.44) (Fig. 4a). The groups with high *NLRC5* expression and low *NLRC5* methylation showed significantly better prognosis than the low *NLRC5* expression group and high *NLRC5* methylation group respectively (HR = 0.45 and HR = 2.31) (Fig. 4a), supporting previous reports that *NLRC5* expression and methylation is correlated with prognosis of melanoma patients^24^. Survival curve analysis of four groups stratified by the level of *NLRC5* expression and mutation load demonstrated that the high *NLRC5* expression/high mutation load group showed better prognosis than the low *NLRC5* expression/low mutation load group (Fig. 4b). Similarly, survival curve analysis for four groups stratified by the level of *NLRC5* promoter methylation and mutation load showed that *NLRC5* methylation high/mutation low group is a high risk group with poor prognosis, and the *NLRC5* methylation low/mutation high group is a lower risk group with better prognosis (Fig. 4b). Taken together, these data indicate that multivariate analysis using *NLRC5* expression/methylation status with mutation load is superior to single variable analysis and may be of value as a prognostic biomarkers in melanoma.

### The expression of NLRC5 and MHC class I-associated genes are correlated with response to anti-PD1 antibody therapy

**Figure 5:**
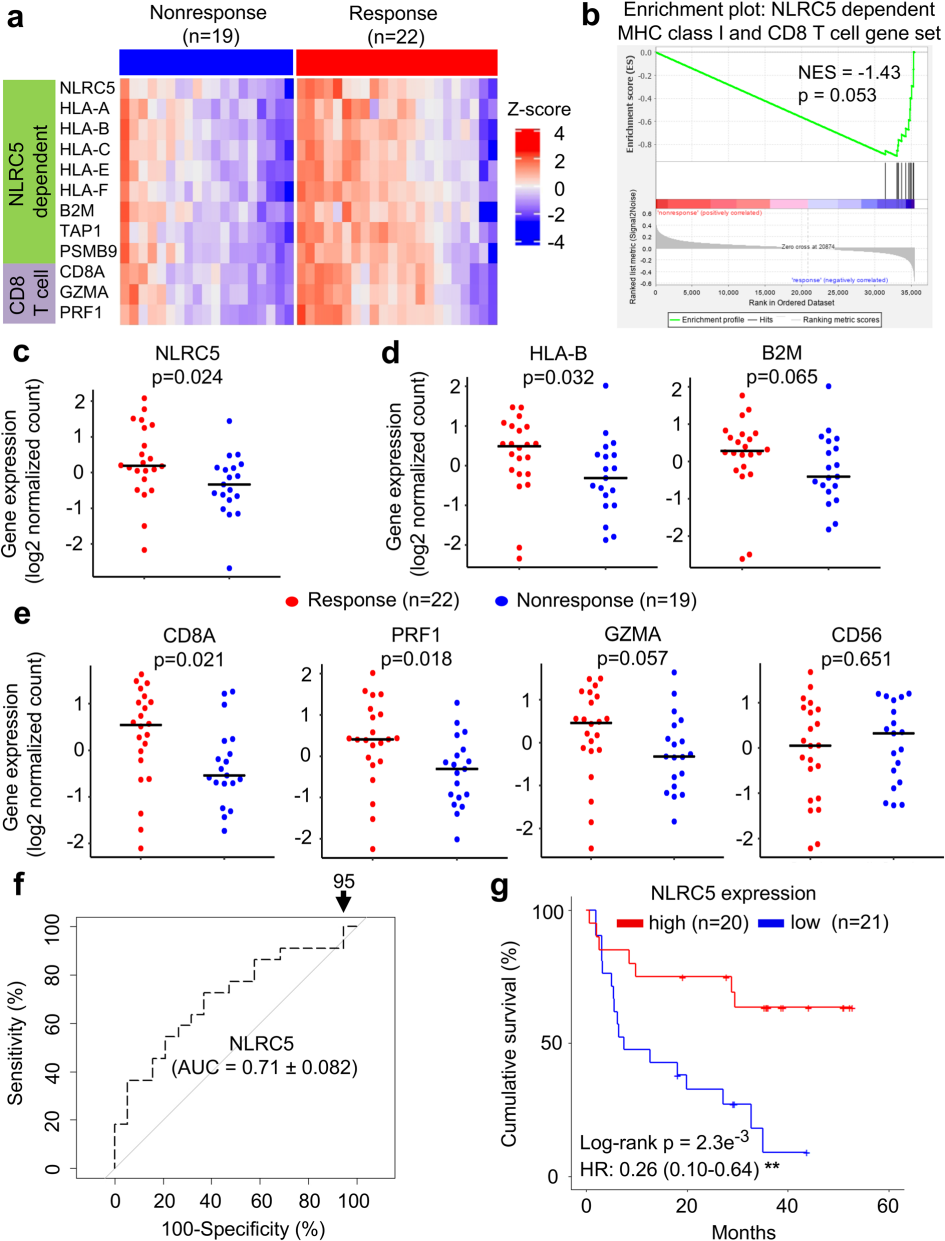
The expression of NLRC5 and NLRC5-dependent MHC class I and CD8+ T cell genes as a predictor to anti-PD1 therapy. Patients groups who benefitted from anti-PD1 therapy (Response, n = 22) and who did not (Nonresponse, n = 19) were analyzed for differential gene set enrichment by (**a**) heatmap and (**b**) GSEA as well as individual gene expression levels of (**c**) *NLRC5*, (**d**) *HLA-B*, *B2M*, (**e**) *CD8A*, granzyme A (*GZMA*), perforin (*PRF1*) and *CD56*. Bar represents the median value. P-values calculated using Mann–Whitney U test. NES, normalized enrichment score. (**f**) ROC curve for logistic regression model using *NLRC5* expression. The numbers with arrow are showing false positive rate with 100% sensitivity. AUC (area under the curve) ± SE (standard error) is depicted. (**g**) Kaplan–Meier estimates of five year overall survival of patients with high and low *NLRC5* gene expression, stratified by median expression (n = 20 and n = 21). Hazard ratio (HR) and 95% confidence interval (CI) was determined by multivariate analysis using Cox regression model (see Methods). **p < 0.01.

Based on our observations of the potential value of NLRC5 as a biomarker in the anti-CTLA4 treatment cohort, we were inspired to extend the analysis to melanoma patient cohorts treated with anti-PD1 therapy. Gene set enrichment analysis indicated that the *NLRC5*-dependent MHC class I and CD8+ T cell gene set was also shown to be reduced in patients who did not respond to anti-PD-1 therapy (Fig. 5a,b). Similar to anti-CTLA4-treated patient cohort, *NLRC5* and *HLA-B* was reduced in non-responders (Fig. 5c), along with a similar trend for *B2M* in anti-PD1-treated melanoma patients (Fig. 5d). Similarly, CD8+ T cell markers, *CD8A*, *PRF1* and *GZMA* were decreased with no change to *CD56* (Fig. 5e). The predictive value of *NLRC5* expression alone in the anti-PD1 cohort was comparable (AUC = 0.71) to what was observed in anti-CTLA4 (Fig. 5f) and NLRC5 expression clearly stratified patients into a high and low overall survival group upon Kaplan–Meier analysis (Fig. 5g). These data indicate that *NLRC5* expression level may also be important for effective response to anti-PD1 monotherapy and may provide predictive information.

## Discussion

Discovery of inhibitory receptors on T cells and development of monoclonal antibodies against them has led to widespread usage of checkpoint blockade therapy in various cancers^2^. Although these therapies are effective for many cancer patients, complete response rate ranges from around 20% for anti-CTLA-4 antibody therapy^42,43^ to 30% for anti-PD/anti-PD-L1 therapy in the case of melanoma^41,43^. These treatments are quite expensive and when ineffective create a significant financial burden on patients and the health care system^44^. Although it is known that the expression of *PD-1*/*PD-L1,* mutation and neoantigen load correlate with responses checkpoint blockade therapy, their predictive power is low, resulting in the treatment of many patients for whom these therapies are ineffective.

This study suggests that *NLRC5* is a biomarker to predict the outcome of CTLA-4 blockade therapy. Since checkpoint therapy relies on T cell activation and *NLRC5* is critical for MHC class I-dependent cytotoxic T cell activation, it is not unexpected that *NLRC5* may play an important role in the response to checkpoint blockade therapy. Indeed, reduced MHC class I immunostaining and gene expression in pretreatment biopsies from anti-CTLA-4 treated melanoma patients predicts resistance to therapy and reduced survival^45^. Similarly, others have independently shown that increased immunoproteasome expression (*PSMB8, PSMB9*) is correlated with better response to anti-CTLA-4 or anti-PD-1 therapy^46^. Although *NLRC5* expression alone has relatively weak predictive power (Fig. 2d), in combination with other variables it yields improved predictive performance. In particular, *NLRC5* expression and neoantigen load/mutation number exhibited a low degree of multi-collinearity and are weakly correlated (Pearson’s coefficient 0.3 and 0.27, respectively, Fig. S3). Combining *NLRC5* expression and mutation numbers demonstrated better AUC values and a lower false positive rate at 100% sensitivity (Fig. 2d). These data indicate that the combination of *NLRC5*/mutation load is superior to these variables alone in identifying non-responders. In contrast to the low correlation between *NLRC5* expression and mutation load, the expression of *CTLA-4*, *PD-1*, *PD-L1* or *PD-L2* relative to *NLRC5* expression carry high to intermediate correlation (Pearson’s coefficient 0.70, 0.83, 0.44 or 0.54, Fig. S3). It appears that *PD-L2* is the best variable to combine with *NLRC5* and mutation number for purposes of prediction (Table S1) and is in line with PD-L2 being a predictive marker in anti-PD-1 checkpoint therapy^47^. Future discovery of other variables will further increase predictive power for response to the checkpoint therapy in melanoma. Although this study involved only a relatively small number of melanoma patients who received anti-CTLA-4 checkpoint therapy, we demonstrated an almost identical relationship between NLRC5 and differentiation of non-responders from responders of anti-PD-1 checkpoint therapy, albeit mutation/neoantigen load data was not available for us to fully replicate our model (Fig. 5). Anti-PD-1/PD-L1 antibody therapy is based on similar mechanisms to increase anti-tumor immune responses. Thus, it is feasible that *NLRC5* expression / mutation load might also be useful for predicting outcomes of other cancer patients treated with anti-PD-1/PD-L1 antibody therapy. Checkpoint blockade therapy was initially tested in melanoma patients, but has been expanded to a dozen cancer types including lung, breast and kidney. Therefore, investigations into the role of *NLRC5* expression and mutation load for the prediction of treatment outcomes in these cancers is of interest.

In summary, this study identified the expression of *NLRC5* as a novel predictive biomarker for immune checkpoint blockade therapies in melanoma. Multivariate analysis using *NLRC5* seems to have significant potential as a predictor of patient response to checkpoint blockade therapy. Validation studies using a larger independent cohort is needed to corroborate these results and to further refine the predictive relationships for checkpoint blockade treatment outcomes.

## Materials and methods

### Data sets

#### Clinical data for anti-CTLA4 therapy

The cohort for the analysis of response to anti-CTLA-4 therapy (ipilimumab) was obtained through Database of Genotypes and Phenotypes (dbGaP)^48,49^, accession number phs000452.v2.p1^38^.

#### TCGA

Data for survival analysis of melanoma was obtained through the Cancer Genome Atlas (TCGA) data portal (https://tcga-data.nci.nih.gov/tcga), Skin Cutaneous Melanoma (SKCM). Gene expression data (mRNASeq; illuminahiseq_rnaseqv2-RSEM_genes_normalized_data), DNA methylation data (humanmethylation450-within_bioassay_data_set_function) and clinical data (Merge_Clinical) were accessed through GDAC Firehose (gdac.broadinstitute.org). Somatic mutation data were accessed through the cBioPortal^50^.

#### Clinical data for anti-PD1 therapy

The raw sequence files for the cohort of response to anti-PD1 therapy was obtained from the European Nucleotide Archive (ENA) under accession PRJEB23709 and matched to clinical data from source study^51^.

### Patient profiling

#### dbGap dataset (anti-CTLA4 therapy)

A patient population of 37 metastatic melanoma patients who had taken ipilimumab monotherapy was analyzed. Patients were stratified by clinical benefit status as described previously^38^. Response to ipilimumab was defined as CR (complete response) or PR (partial response) by Response Evaluation Criteria in Solid Tumors (RECIST) criteria or SD (stable disease) by RECIST criteria^52^ with overall survival greater than 1 year. Non-response to ipilimumab was defined as PD (progressive disease) or SD (stable disease) by RECIST criteria with overall survival less than 1 year.

#### ENA dataset (anti-PD1 therapy)

A patient population of 41 melanoma patients treated with anti-PD1 therapy using either nivolumab (n = 9) or pembrolizumab (n = 32) with available pre-treatment sampled tumor RNA-seq data was analyzed. Patient stratification into responder and non-responders was performed under the same criteria used with the anti-CTLA-4 patients.

### Gene expression analysis

RNA sequences were downloaded and converted to FastQ file format using SRA Toolkit v2.6.3. Paired-end reads were checked to trim for low quality bases and adaptor sequences using Trimmomatic^53^. For the dbGap dataset, filtered reads were mapped to the GRCh37/hg19 assembly using TopHat v2.0.13^54^ and HTSeq^55^ was then used to generate raw read counts per gene using intersection-nonempty parameter to account for ambiguous read mappings. For the ENA dataset, filtered reads were mapped to the GRCh38/hg19 assembly using HISAT2^56^ with GNU-parallel^57^ and BAM files indexed and sorted by SAMtools^58^. featureCounts^59^ was used to count reads mapped to each gene. Gene expression values were generated for further analyses using DESeq2^60^, following recommended guidelines by the authors. The expression levels of *NLRC5*, *HLA-B*, *B2M*, *CD8A*, *granzyme A* (*GZMA*), *perforin* (*PRF1*) and *CD56* measured by RNA sequencing (RNA-seq) were compared between responders and non-responders using the Mann–Whitney U Test.

### Gene set enrichment analysis (GSEA)

Gene Set Enrichment Analysis (GSEA, http://www.broad.mit.edu/gsea/) ^61^ was used to assess differential expression of *NLRC5*-dependent MHC class I and CD8+ T cell gene set between response and non-response group to anti-CTLA4 or anti-PD1 therapy. The gene set tested was based on knowledge of the literature concerning *NLRC5* and the MHC class I antigen presentation pathway^23,24,37^.

### Mutation and neoantigen analysis

For analyses involving numbers of mutation and neoantigen (mutation load and neoantigen load, respectively), 35 of the 37 melanoma patients treated with ipilimumab had data available^38^. Those were compared between responders and non-responders to ipilimumab using the Mann–Whitney U Test. The bidimensional combination of NLRC5 expression and mutation or neoantigen load was assessed and p-values were calculated using Hotelling’s T^2^ Test to compare between responder and non-responder to ipilimumab. Next, to evaluate the influence of those variables for response to ipilimumab, cohort was divided into four groups based on the level of NLRC5 expression and mutation or neoantigen load and was calculated for the response rate to ipilimumab. Patients carrying higher value of the median are defined as high group, those carrying lower value of the median are defined as low group in respective variables. Statistical significance between the groups of high NLRC5 expression/high mutation or neoantigen load and low NLRC5 expression/low mutation or neoantigen load was determined by the χ2 test.

Expression values for other genes known as predictors of response to anti-CTLA-4 therapies, such as *CTLA-4*, *PD-1*, *PD-L1* and *PD-L2* were combined with *NLRC5* expression and mutation or neoantigen load and represented in a three-dimensional scatterplot.

### Logistic regression analysis

Logistic regression models were fitted with different combinations of the following covariates: the values for expression of 5 genes (*NLRC5*, *PD-L1*, *PD-L2*, *CTLA-4* and *PD-1*), mutation load, and neoantigen load. Up to 3 of these variables were considered for the regression models at a time. Samples with missing values were eliminated before fitting the regression. Multicollinearity was assessed through calculation of variance inflation factors and Pearson’s correlation coefficient (Fig. S3). A scatterplot matrix was created with fitted curves and regression lines and the distribution of each variable was inspected. An ROC curve was generated for each combination of covariates and AUC ± standard error (SE) generated using the pROC package (version 1.8)^62^ in R. The training data was used as the prediction data for the ROC curves. Threshold values were determined at points where the sensitivity is 100%. These curves were plotted and a selection were reported. A bootstrapping procedure with 10,000 repetitions was used to estimate 95% confidence intervals for the curves as well as calculate a mean AUC. This was accomplished by sampling the cohort with replacement to create new groupings of data (the same size as the original cohort) then used to construct ROC curves. The AUC was calculated for each of these new curves. The confidence interval was determined by ordering the AUCs by value and returning the value at the index 2.5% of the length of the list away from the beginning and end. The best prediction model was chosen based on the highest mean AUC.

### Survival analysis

Survival analysis was performed on a cohort of melanoma patients obtained from TCGA (n = 319) or ENA (n = 41). The TCGA cohort was filtered to only include patients who had had all three data types available (RNA-seq, mutation load and methylation) as well as clear cancer tumor stage record. *NLRC5* methylation was assessed as the sum of the methylation of probe sites (cg08159663, cg07839457 and cg16411857) that negatively correlated with *NLRC5* gene expression (Fig. S4). Survival curves were calculated using the Kaplan–Meier method depicting the difference in survival between the groups through division of the cohort into top and bottom 50% based on median *NLRC5* expression, *NLRC5* methylation or mutation load. Patients were stratified in a similar fashion by two variables (*NLRC5* expression and mutation load) yielding four groups (high *NLRC5* expression/high mutation load, high/low, low/high, low/low, respectively). The same was also performed for *NLRC5* methylation and mutation load (high *NLRC5* methylation/high mutation load, high/low, low/high, low/low, respectively). For univariate survival curves, statistical significance was assessed by log-rank test and for multivariate survival curves, pairwise log-rank test with Benjamini–Hochberg FDR correction was used. A Cox proportional hazard regression model was used to evaluate the effects of age and cancer stage as additional covariates. Cancer stage was not available for patients in the anti-PD-1 dataset.

### Statistical analysis

Statistical analysis was performed using R 3.6.0 and RStudio 1.1.463.

### Ethical approval and ethical standards

The data used in our study were obtained from public databases, therefore, ethical approval was not required.

## Supplementary Information

Supplementary Information 1.

Supplementary Video 1.

Supplementary Video 2.

Supplementary Video 3.

Supplementary Video 4.

Supplementary Video 5.

Supplementary Video 6.

Supplementary Video 7.

Supplementary Video 8.
