# Supplementary material for: NLRC5/CITA expression correlates with efficient response to checkpoint blockade immunotherapy
Source: Sci Rep. 2021 Feb 5;11:3258. doi: 10.1038/s41598-021-82729-9 (PMC7865024; doi:10.1038/s41598-021-82729-9)
Supplement: Supplementary file 1 — Supplementary Information 1. [file 41598_2021_82729_MOESM1_ESM.docx]

Supplementary Data

Title: NLRC5/CITA expression correlates with efficient response to checkpoint blockade immunotherapy

**Authors:** Sayuri Yoshihama^1,2^, Steven X. Cho^3^, Jason Yeung^1^, Xuedong Pan^4^, Gregory Lizee^5^, Kranti Konganti^6^, Valen E. Johnson^4^ and Koichi S. Kobayashi^1,3*^

Correspondence to: kobayashi@medicine.tamhsc.edu

**This PDF file includes:**

Figs. S1 to S4

Tables S1

Captions for Movies S1 to S8

**Other Supplementary Materials for this manuscript include the following:**

Movies S1 to S8


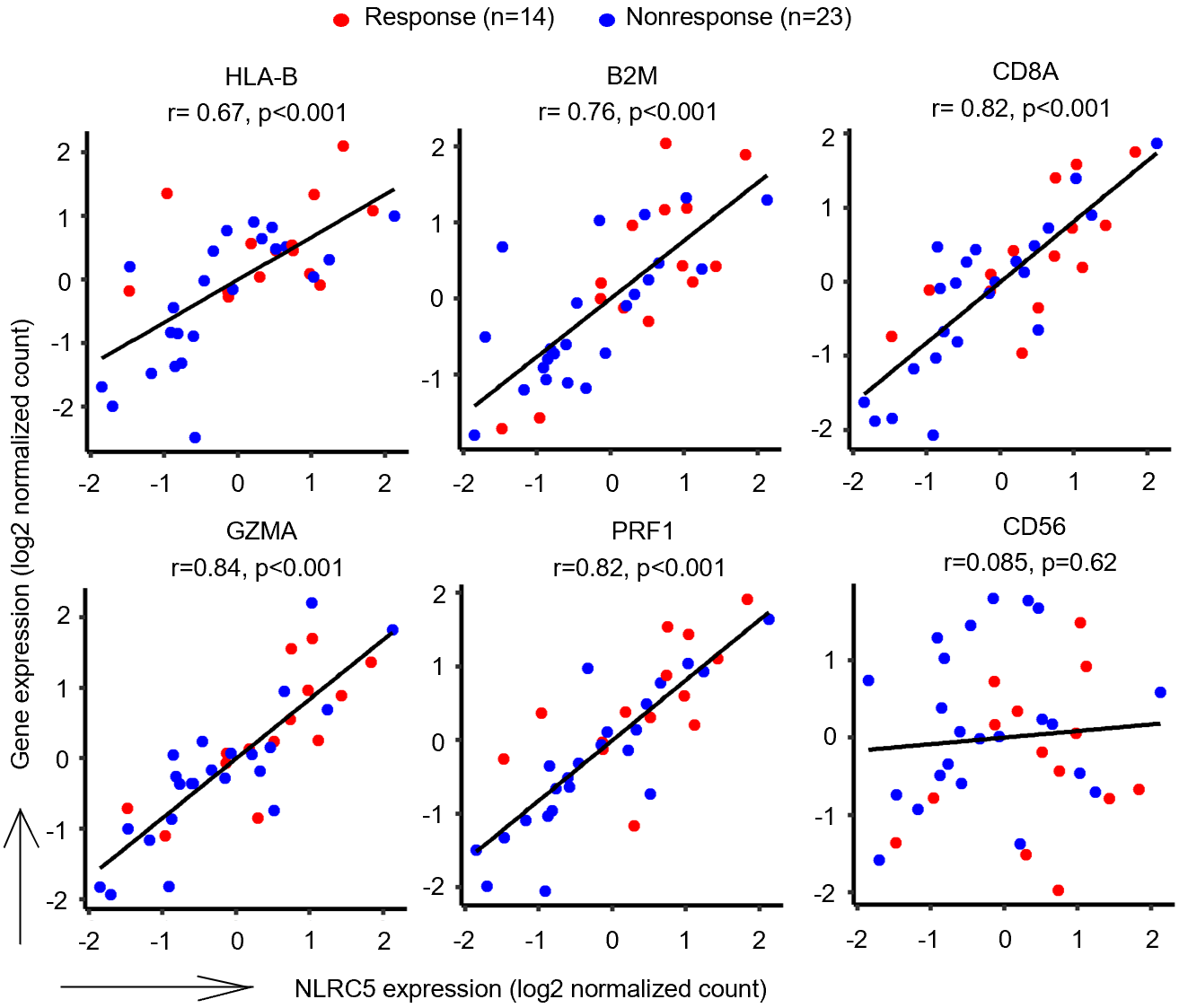


**Figure S1.**

***NLRC5* expression is correlated with expression of MHC class I associated genes in the melanoma patient cohort.**

Scatterplots for expression of *NLRC5* and indicated genes in Response (n=14) and Nonresponse (n=23) groups in the melanoma patient cohort treated with anti-CTLA4 therapy. Pearson’s correlation coefficient (r) and associated p-values are indicated.


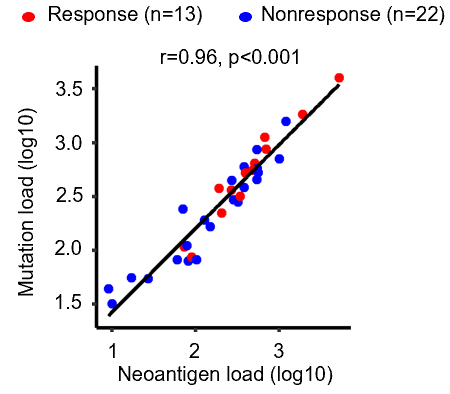


**Figure S2.**

**Mutation and neoantigen load are highly correlated in anti-CTLA4 treated melanoma.**

Scatterplot for mutation and neoantigen load in Response (n=13) and Nonresponse (n=22) groups. Pearson’s correlation coefficient (r) and associated p-values are indicated.


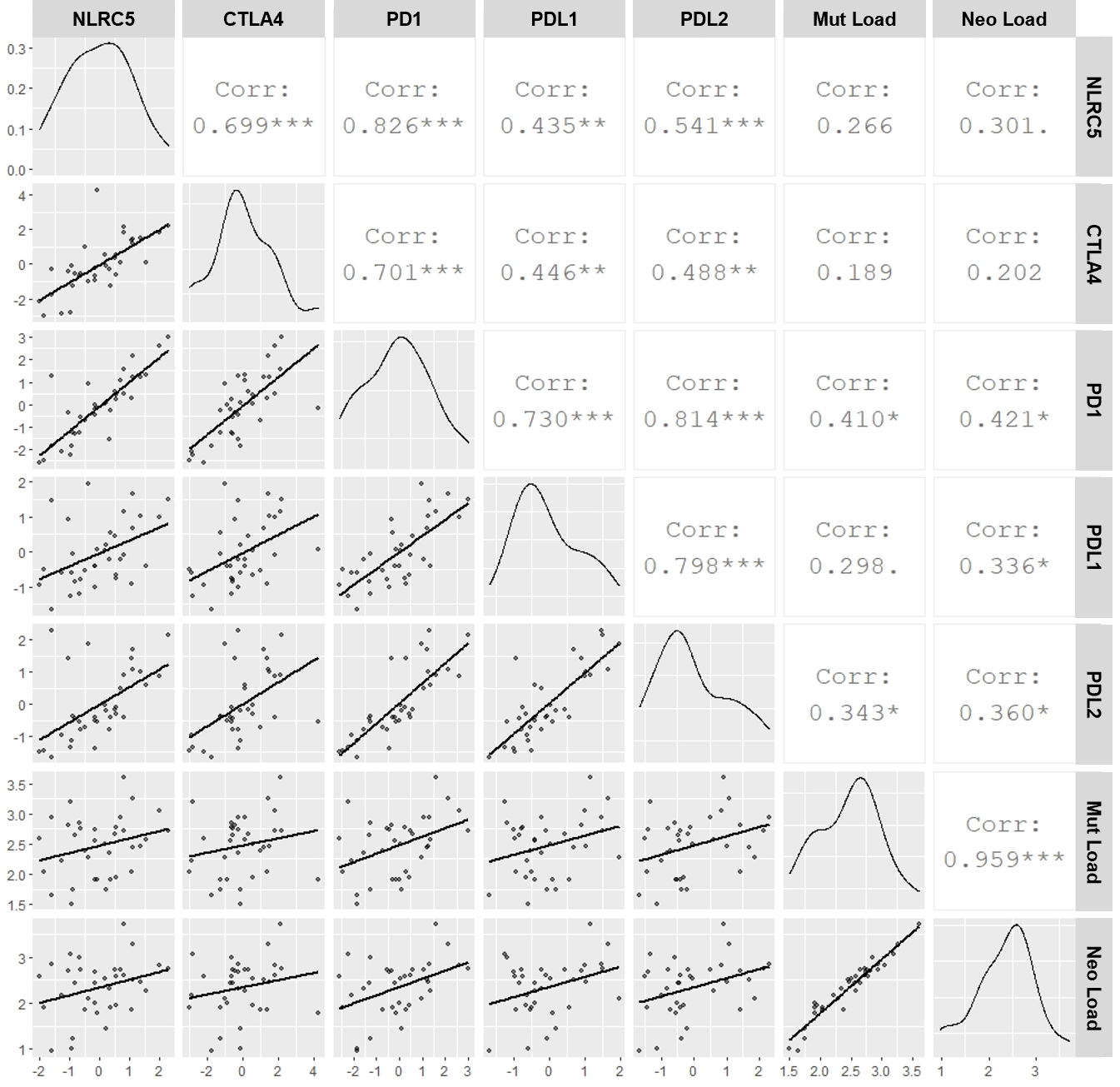


Figure S3

**Multicollinearity between variables in melanoma patient cohort treated with anti-CTLA4 therapy.**

Scatterplot matrix (lower panel) to detect multicollinearity between variables, including log2 normalized gene expression of *NLRC5, CTLA-4, PD-1, PD-L1* and *PD-L2*, mutation load (log10) and neoantigen load (log10), considered for logistic regression model. Upper panels depict the Pearson’s correlation coefficient (r) with associated p-value depicted as *, p<0.05; **, p<0.01; ***, p<0.001.


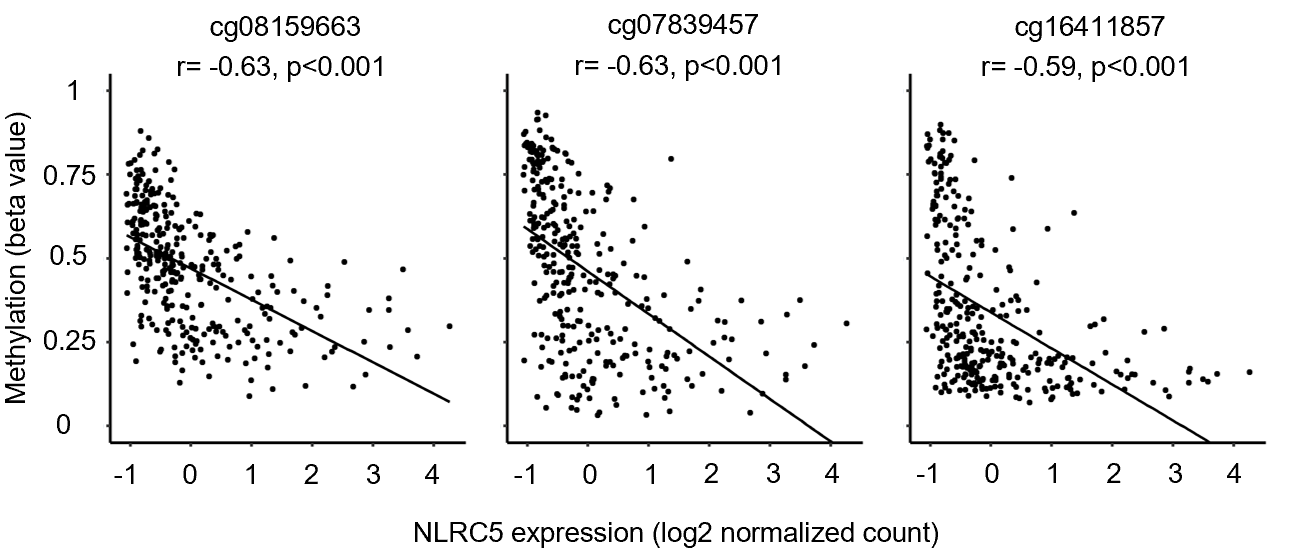


Figure S4.

**DNA-methylation in the *NLRC5* promoter in melanoma patient cohort is negatively correlated with NLRC5 gene expression.**

Scatterplots for *NLRC5* expression and DNA methylation detected by three indicated methylation-specific probes in the TCGA cohort (n=319). Spearman’s correlation coefficient (r) and associated p-values are indicated.


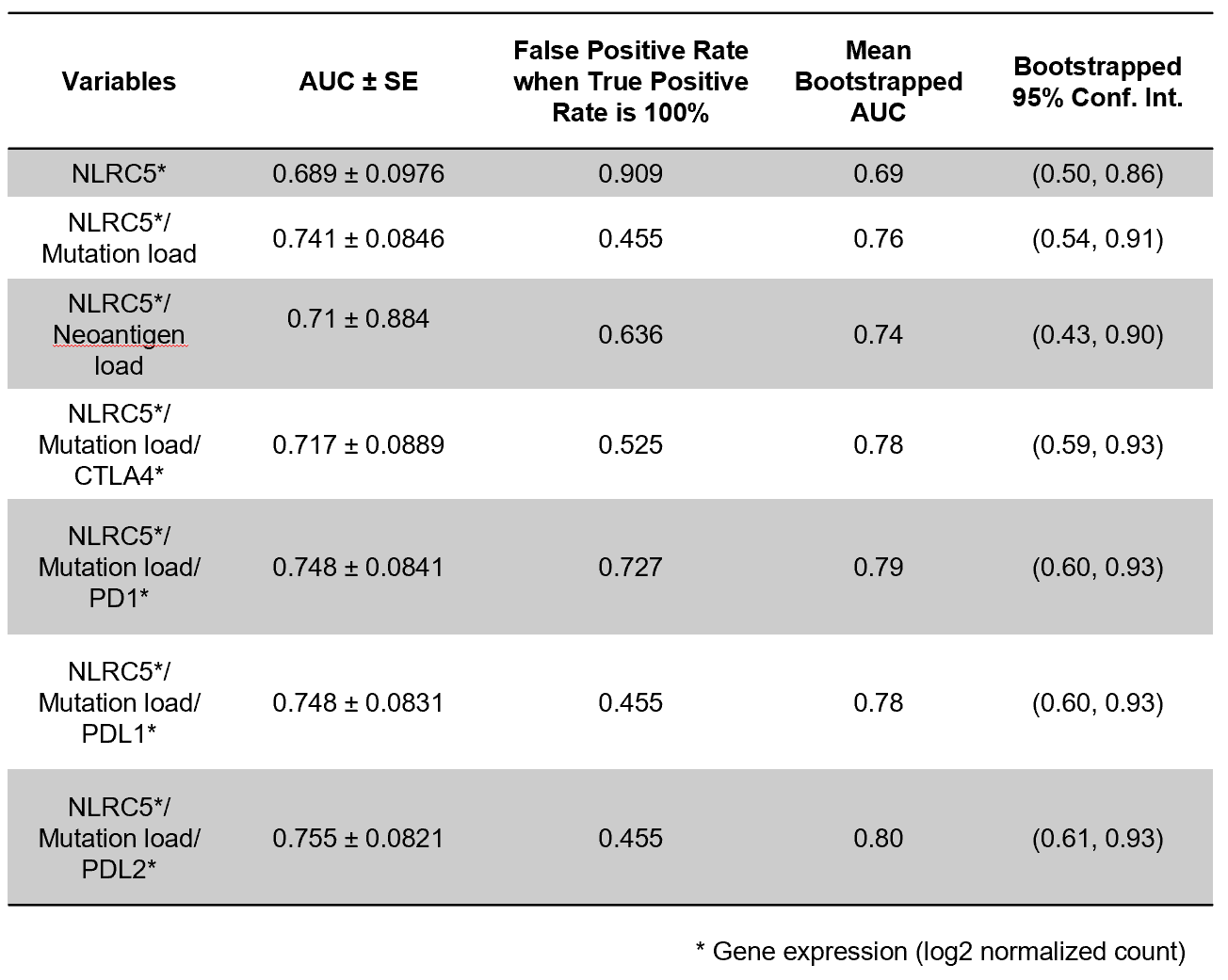


**Table S1.**

AUC values and corresponding false positive rate in different combinations of variables in the melanoma patient cohort treated with anti-CTLA4 therapy.

Movie S1.

A scatterplot based on NLRC5 expression, mutation load, and CTLA4 expression with 95% CI ellipsoids. Responding patients are marked in red with nonresponding patients marked in blue.

Movie S2.

A scatterplot based on NLRC5 expression, mutation load, and PD1 expression with 95% CI ellipsoids. Responding patients are marked in red with nonresponding patients marked in blue.

Movie S3.

A scatterplot based on NLRC5 expression, mutation load, and PDL1 expression with 95% CI ellipsoids. Responding patients are marked in red with nonresponding patients marked in blue.

Movie S4.

A scatterplot based on NLRC5 expression, mutation load, and PDL2 expression with 95% CI ellipsoids. Responding patients are marked in red with nonresponding patients marked in blue.

Movie S5.

A scatterplot based on NLRC5 expression, neoantigen load, and CTLA4 expression with 95% CI ellipsoids. Responding patients are marked in red with nonresponding patients marked in blue.

Movie S6.

A scatterplot based on NLRC5 expression, neoantigen load, and PD1 expression with 95% CI ellipsoids. Responding patients are marked in red with nonresponding patients marked in blue.

Movie S7.

A scatterplot based on NLRC5 expression, neoantigen load, and PDL1 expression with 95% CI ellipsoids. Responding patients are marked in red with nonresponding patients marked in blue.

Movie S8.

A scatterplot based on NLRC5 expression, neoantigen load, and PDL2 expression with 95% CI ellipsoids. Responding patients are marked in red with nonresponding patients marked in blue.
